# Morphological and quantitative CT features of anterior mediastinal lesions in TAFRO syndrome and idiopathic multicentric Castleman disease

**DOI:** 10.3389/fimmu.2025.1656489

**Published:** 2025-09-15

**Authors:** Lamiaa Mohamed, Masataka Umeda, Shin Tsutsui, Ryo Toya, Ayaka Umetsu, Mizuna Otsuka, Yushiro Endo, Toshimasa Shimizu, Shoichi Fukui, Remi Sumiyoshi, Atsushi Kawakami, Tomohiro Koga

**Affiliations:** ^1^ Department of Immunology and Rheumatology, Nagasaki University Graduate School of Biomedical Sciences, Nagasaki, Japan; ^2^ Leading Medical Research Core Unit, Nagasaki University Graduate School of Biomedical Sciences, Nagasaki, Japan; ^3^ Department of Radiology, Nagasaki University Graduate School of Biomedical Sciences, Graduate School of Biomedical Sciences, Nagasaki University, Nagasaki, Japan

**Keywords:** TAFRO syndrome, idiopathic multicentric Castleman disease, anterior mediastinal lesion, IgG4 - related disease, computed tomography

## Abstract

We investigated the diagnostic challenges of TAFRO syndrome and idiopathic multicentric Castleman disease (iMCD), focusing on the usefulness of anterior mediastinal lesions for distinguishing the disease subtypes. A comparative analysis using computed tomography (CT) imaging was performed for three patient groups: TAFRO without iMCD/iMCD-TAFRO (n=13), iMCD-idiopathic plasmacytic lymphadenopathy (IPL)- not otherwise specified (NOS) (n=16), and IgG4-related disease (IgG4-RD) (n=59). Lesions were categorized into increased fat density, micronodular opacity, and mass. The lesions’ CT attenuation values were compared, and receiver operating characteristic (ROC) curve analyses assessed their diagnostic relevance. Anterior mediastinal lesions were most frequent in the TAFRO without iMCD/iMCD-TAFRO group (85%) compared to the iMCD-IPL/NOS group (31%) and IgG4-RD group (6.8%). Distinct patterns such as increased fat density were predominantly observed in the TAFRO without iMCD/iMCD-TAFRO group. The CT values showed significant intergroup differences, with ROC analyses confirming high diagnostic accuracy for distinguishing the TAFRO without iMCD/iMCD-TAFRO group from the other groups. Post-treatment, all patients with TAFRO without iMCD/iMCD-TAFRO showed improvement in CT readings, whereas only half of the patients with iMCD-NOS group showed changes. These findings emphasize the importance of CT-detected anterior mediastinal lesions in the diagnosis of TAFRO without iMCD/iMCD-TAFRO and warrant further research to validate these results.

## Introduction

TAFRO syndrome (characterized by thrombocytopenia, anasarca, fever, reticulin fibrosis, and organomegaly) and idiopathic multicentric Castleman disease (iMCD) pose considerable diagnostic challenges due to its clinical features that overlap with those of other conditions and the lack of specific diagnostic markers (1). In recent years, clinical and pathological analyses have led to the recognition of four related subtypes: iMCD with idiopathic plasmacytic lymphadenopathy (iMCD-IPL), iMCD not otherwise specified (iMCD-NOS), iMCD with TAFRO syndrome (iMCD-TAFRO), and TAFRO syndrome without iMCD-consistent lymph node histology (TAFRO without iMCD) (2–4). iMCD-IPL is characterized by polyclonal hypergammaglobulinemia and marked infiltration of mature plasma cells within lymph nodes, typically presenting with a chronic or indolent clinical course that responds well to interleukin-6 (IL-6) blockade and is associated with a generally favorable prognosis (3, 4). In contrast, iMCD-NOS includes cases that do not meet the criteria for either iMCD-IPL or iMCD-TAFRO, often exhibiting mixed histopathologic patterns with variable clinical behavior and treatment responsiveness (3, 4). iMCD-TAFRO is a clinically aggressive subtype defined by the coexistence of iMCD-consistent lymph node histology and TAFRO syndrome, a clinical constellation that includes thrombocytopenia, anasarca, fever, reticulin myelofibrosis, renal dysfunction, and organomegaly. Histologically, iMCD-TAFRO typically shows a hypervascular pattern with atrophic germinal centers and proliferation of endothelial venules (4, 5). This subtype frequently presents with acute or subacute onset, progresses rapidly, and often exhibits limited responsiveness to IL-6 inhibition, resulting in a poorer prognosis than other iMCD variants (2). TAFRO without iMCD refers to patients who meet clinical criteria for TAFRO syndrome but lack lymph node findings consistent with iMCD. Although not formally classified as iMCD, these patients demonstrate a clinical phenotype nearly indistinguishable from iMCD-TAFRO, including severe systemic inflammation and multiorgan involvement (2). Despite the use of therapeutic strategies such as the use of glucocorticoids, B-cell depletion, and an IL-6 signaling blockade, TAFRO syndrome remains a highly fatal condition with a considerable proportion of treatment-refractory cases (6), highlighting the urgent need for early diagnosis to prevent disease progression and improve clinical outcomes.

Case reports have described anterior mediastinal abnormalities in iMCD and in TAFRO syndrome, with the histopathological assessment in some cases identifying lymphocytic and plasmacytic infiltration into the anterior mediastinal adipose tissues (7–12). The authors of some of these reports noted that anterior mediastinal lesions showed improvement in TAFRO syndrome following treatment (7, 9, 10), and another report indicated that the characteristics of these lesions may change over time (12). The prevalence of anterior mediastinal lesions has been reported in only a few studies (13, 14), but those studies did not include the evaluation of tissue density through computed tomography (CT) values, and there is a lack of longitudinal studies assessing the impact of treatment on anterior mediastinal lesions. Based on the above, attention to anterior mediastinal abnormalities on CT has been recognized as important in the diagnosis of iMCD and TAFRO syndrome; however, it remains unclear whether these abnormalities follow similar or distinct courses across the highly heterogeneous subgroups within these disease entities. To address this gap, we conducted the present study to (*i*) investigate how to differentiate anterior mediastinal lesions in both iMCD and TAFRO syndrome, and (*ii*) explore the potential of CT values in the diagnoses of these diseases, as well as the impact of treatment on lesion improvement. Understanding the characteristics of anterior mediastinal lesions through CT scans is crucial for the early diagnosis of TAFRO syndrome, which is a rapidly progressive disease.

## Patients and methods

### Study design and patients

We conducted a comparative descriptive analysis of the cases of patients who had been diagnosed with iMCD, TAFRO syndrome, or IgG4-related disease (IgG4-RD) at Nagasaki University Hospital between 2005 and 2024. As a lymphoproliferative disorder frequently considered in the differential diagnosis of iMCD/TAFRO, IgG4-RD was used as a control group in this study (15). Patients who met the 2017 international consensus criteria for iMCD (16), the 2019 Japanese diagnostic criteria for TAFRO syndrome (17), and the 2020 revised comprehensive diagnostic criteria for IgG4-RD (18) were enrolled. Three patient groups were evaluated: TAFRO without iMCD/iMCD-TAFRO (n=13), iMCD-idiopathic plasmacytic lymphadenopathy (IPL)/not otherwise specified (NOS) (n=16) as defined (2, 3, 19), and a control group consisting of patients with IgG4-RD (n=59). The patients’ clinical features and laboratory test data, including CHAP score (20) and TAFRO syndrome severity score (17), were gathered from the medical records.

### CT examination

**Figure 1:**
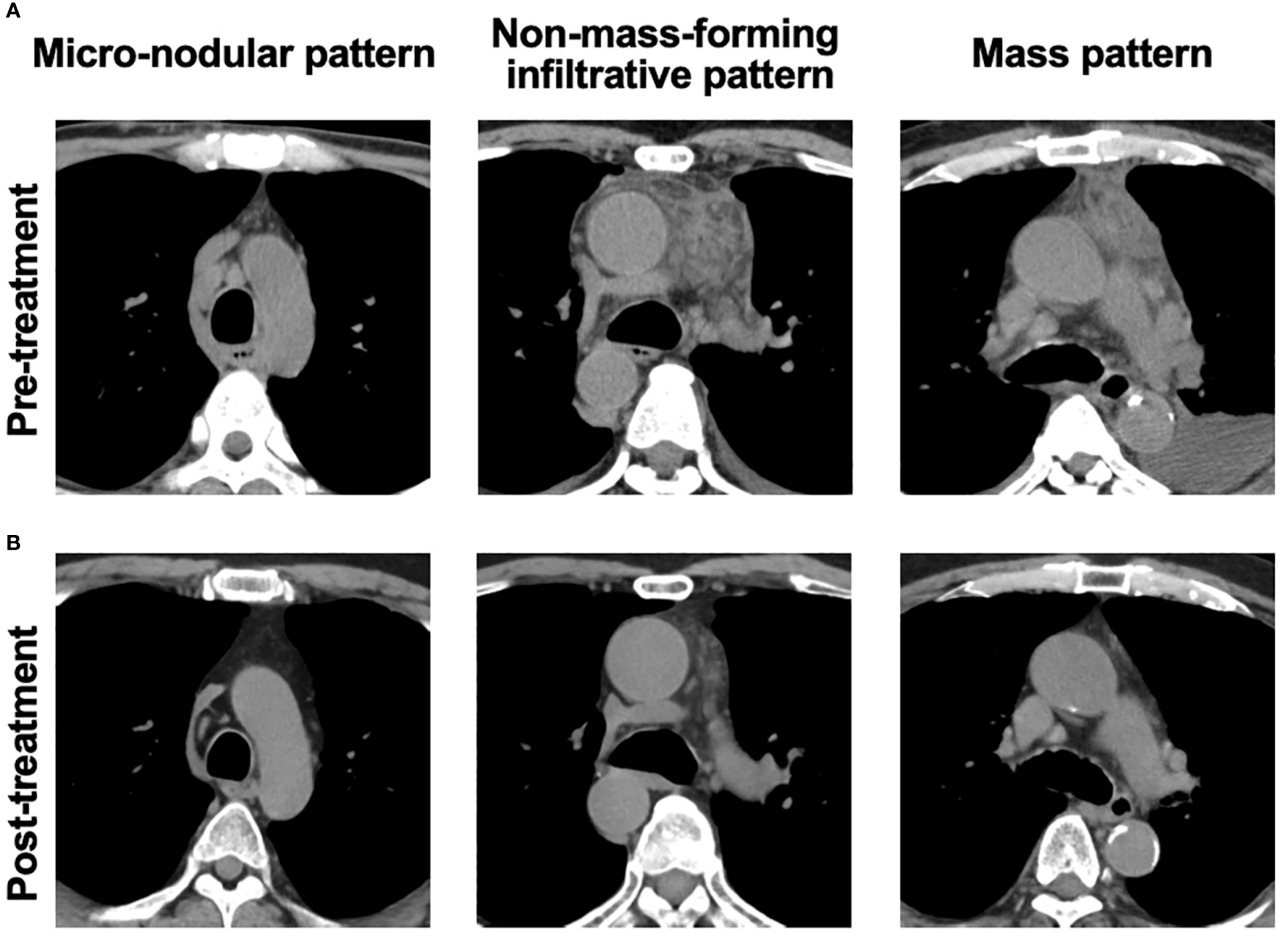
Representative images of abnormalities in anterior mediastinum lesions. **(A)** Representative pre-treatment CT images showing three types of anterior mediastinal abnormalities: the micro-nodular pattern (idiopathic multicentric Castleman disease-not otherwise specified [iMCD-NOS]), the non-mass-forming infiltrative pattern (idiopathic multicentric Castleman disease-TAFRO subtype [iMCD-TAFRO]), and the mass pattern (TAFRO syndrome). **(B)** Post-treatment CT images showing improvement in each respective abnormality.

Plain CT imaging (without contrast) was used to assess anterior mediastinal lesions, determining the presence or absence of abnormalities and classifying them into three distinct patterns with partial reference to the previous report (13): the micro-nodular pattern, the non-mass-forming infiltrative patten, and the mass patten (representative images are provided in **Figure 1A**).

Two observers evaluated the images, and Cohen’s kappa statistics were used to assess the inter-observer agreement between the two evaluators for the anterior mediastinal lesions and their subtypes. The overall agreement for the presence or absence of anterior mediastinal lesions was excellent (κ = 0.938). The kappa values for individual radiological features were 0.793 for ‘increase in fat density,’ 0.725 for ‘micro-nodular opacity,’ and 1.000 for ‘mass,’ indicating substantial-to-perfect agreement across the evaluated features. In cases in which the two evaluators disagreed, the final classification was determined through consensus discussion.

For a quantitative evaluation, the CT attenuation value was measured by placing a region of interest (ROI) in the anterior mediastinum at the level showing the largest cross-sectional diameter. We compared the first and last scans (taken >1 year apart), focusing on the CT attenuation values across the different groups.

### Statistical analyses

Continuous variables were compared with Wilcoxon’s rank sum test, and categorical variables were analyzed with Fisher’s exact test. The Kruskal-Wallis test was used for comparisons of three groups. When significant differences were identified, multiple comparisons were performed using Dunn’s test to account for multiple testing. Changes in the anterior mediastinal lesions from before to after treatment were evaluated with a non-parametric paired test (Wilcoxon signed-rank test).

A receiver operating characteristic (ROC) curve was created to assess the diagnostic utility of CT values for distinguishing TAFRO without iMCD/iMCD-TAFRO from iMCD-NOS or IgG4-RD. Areas under the curve (AUCs) were internally validated using bootstrap resampling (n=1,000) and logistic regression on each dataset. Statistical significance for all tests was defined as a two-tailed p-value <0.05.

The statistical analyses were performed using GraphPad Prism (GraphPad Software, San Diego, CA), and JMP Pro 17.0 Statistical Software (SAS Institute, Cary, NC).

## Results

### The prevalence of anterior mediastinal lesions in iMCD and TAFRO syndrome

The patients’ demographic and laboratory findings are summarized in **Supplementary Table S1**. The clinical manifestations of TAFRO syndrome—including pleural effusion, pericarditis, ascites, edema, renal dysfunction, fluid retention, hepatosplenomegaly, and thrombocytopenia—are summarized in **Supplementary Table S2**. Anterior mediastinal lesions were significantly more prevalent in the TAFRO without iMCD/iMCD-TAFRO group, in which 85% of the patients had these lesions, compared to 31% in the iMCD-IPL/NOS group and only 6.8% in the IgG4-RD group (**Table 1**). Of the patients in the TAFRO without iMCD/iMCD-TAFRO group, 82% exhibited non-mass-forming infiltrative lesions, and 18% showed mass lesions. In contrast, all of the lesions in the iMCD-IPL/NOS and IgG4-RD groups exhibited the micro-nodular pattern. No significant differences in the presence or characteristics of anterior mediastinal lesions, including CT attenuation values, were observed between the iMCD-TAFRO and TAFRO without iMCD groups (**Supplementary Table S3**). The presence and CT attenuation values of anterior mediastinal lesions were also compared between groups stratified by lymph node histopathological findings, and no significant differences were observed (**Supplementary Table S4**).

**Table 1:** Patient demographics and characteristics.

Variable	IgG4-RD n=59	iMCD-IPL/NOS n=16	TAFRO without iMCD/iMCD-TAFRO n=13)	p-value
Age, yrs	62 (55, 69)	52 (44, 66)	63 (47, 73)	0.3
Females	18 (31%)	10 (63%)	4 (31%)	0.056
Any abnormalities in anterior mediastinal lesions	4 (6.8%)	5 (31%)	11 (85%)	<0.001
Non-mass-forming infiltrative lesions	0 (0%)	0 (0%)	9 (69%)	<0.001
Micro-nodular lesions	4 (6.8%)	5 (31%)	0 (0%)	0.007
Mass lesions	0 (0%)	0 (0%)	2 (15%)	0.003
CT value, HU	−88 (−100, −71)	−69 (−94, −44)	−28 (−34, −12)	<0.001

Values are n (%) of patients or the median (Q1, Q3). HU, Hounsfield unit; IgG4-RD, IgG4-related disease; iMCD, idiopathic multicentric Castleman disease; IPL, idiopathic plasmacytic lymphadenopathy; NOS, not otherwise specified; TAFRO, thrombocytopenia, anasarca, fever, reticulin fibrosis, organomegaly.

### The clinical importance of the anterior mediastinal CT values in iMCD and TAFRO syndrome

**Figure 2:**
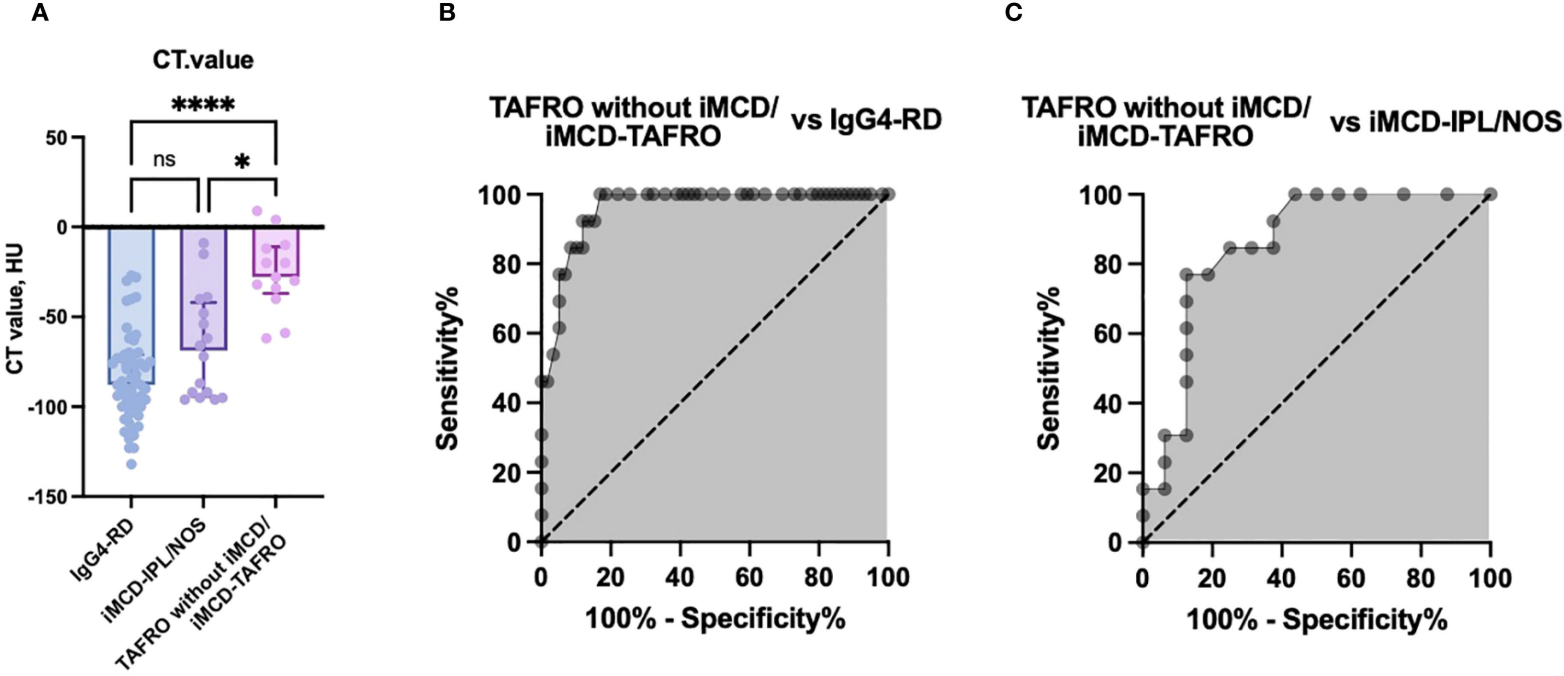
The CT attenuation values of anterior mediastinal lesions and receiver operating characteristic (ROC) curve analyses for disease differentiation. **(A)** Comparison of CT attenuation values (HU) in anterior mediastinal lesions among the TAFRO without iMCD/iMCD-TAFRO group, the iMCD-IPL/NOS group, and the IgG4-RD group. B,C: ROC curve analyses of CT attenuation values to distinguish TAFRO without iMCD/iMCD-TAFRO from IgG4-RD **(B)** and from iMCD-IPL/NOS **(C)**. *p<0.05, ***p<0.001, ns: not significant. *Bars:* median values. *Error bars:* interquartile range (IQR). AUC, area under the curve; HU, Hounsfield units; IgG4-RD, immunoglobulin G4-related disease; iMCD, idiopathic multicentric Castleman disease; IPL, idiopathic plasmacytic lymphadenopathy; NOS, not otherwise specified.

The CT attenuation values were significantly different between the TAFRO without iMCD/iMCD-TAFRO group (median −28 HU, interquartile range [IQR] −34 to −12) and both the iMCD-IPL/NOS group (median −69 HU, IQR −94 to −44; p<0.01) and the IgG4-RD group (median −88 HU, IQR −100 to −71; p<0.001) (**Figure 2A**). To investigate the usefulness of the CT value of the mediastinal lesions for the diagnoses of iMCD and TAFRO syndrome, we then generated ROC curves for the CT values to distinguish the patients with TAFRO without iMCD/iMCD-TAFRO from those with iMCD-IPL/NOS or IgG4-RD. The results of the ROC analyses indicated high diagnostic accuracy, with an AUC of 0.95 for distinguishing TAFRO patients without iMCD/iMCD-TAFRO from those with IgG4-RD (cut-off: −62 HU, sensitivity: 100%, specificity: 81.4%) and an AUC of 0.86 for differentiating TAFRO without iMCD/iMCD-TAFRO from iMCD-IPL/NOS (cut-off −34 HU, sensitivity 76.9%, specificity 87.5%) (**Figures 2B, C**).

The internal validation using the bootstrap method yielded robust AUC values, confirming the high diagnostic performance of CT values for differentiating the disease subtypes. The mean AUC values from the bootstrap analysis were as follows: 0.97 for distinguishing patients with TAFRO without iMCD/iMCD-TAFRO from those with IgG4-RD and 0.92 for differentiating TAFRO without iMCD/iMCD-TAFRO from iMCD-IPL/NOS. These values were comparable to the original ROC analysis results, indicating high internal consistency and reliability of the diagnostic model.

### Longitudinal CT assessment of anterior mediastinal abnormalities

**Figure 3:**
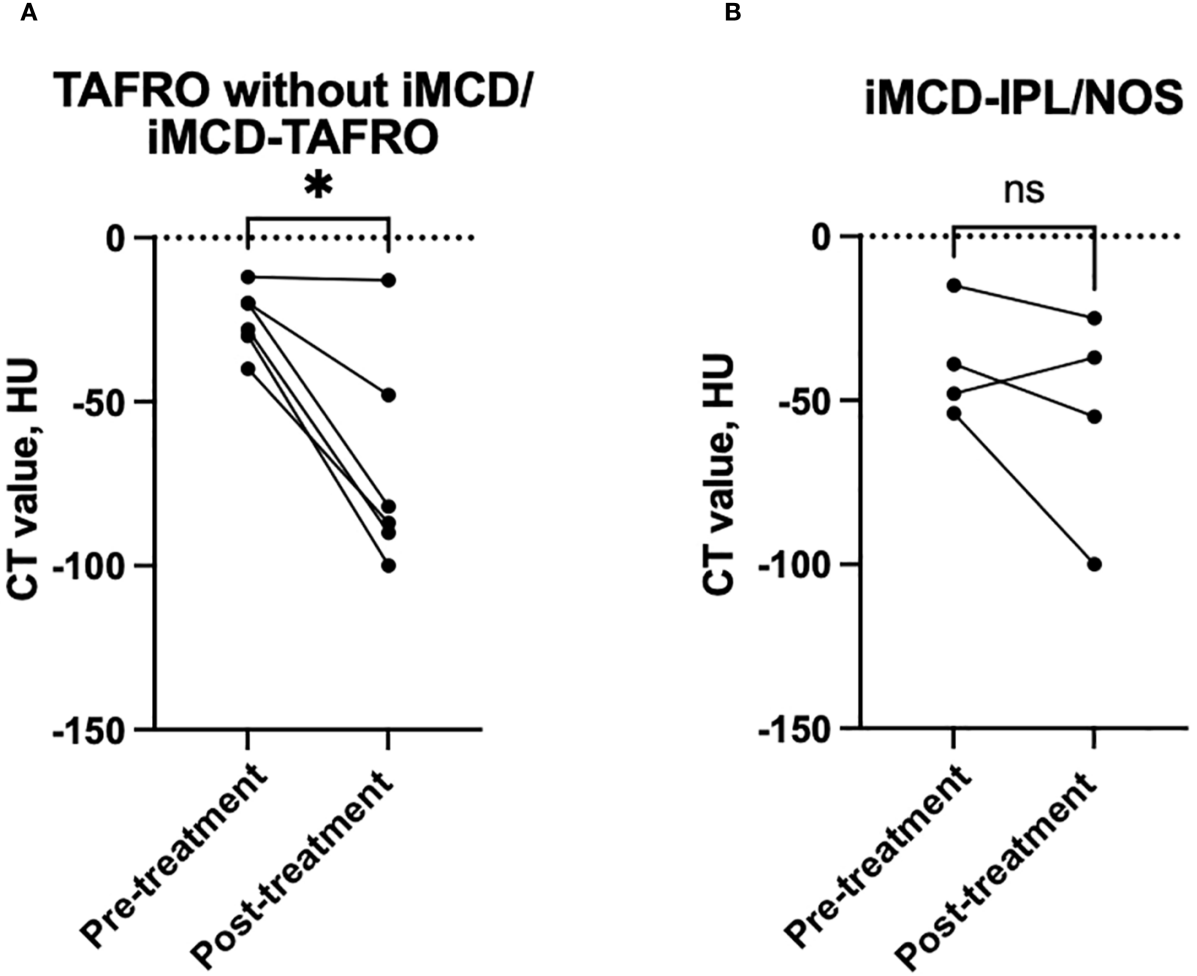
Longitudinal changes in the anterior mediastinal CT attenuation values from before to after treatment. **(A)** In the patients with TAFRO without iMCD or with iMCD-TAFRO, the anterior mediastinal CT values significantly decreased after treatment. **(B)** In contrast, no significant change was observed in the iMCD-IPL/NOS group. Each line represents an individual patient. *p<0.05; ns: not significant. iMCD, idiopathic multicentric Castleman disease; IPL, idiopathic plasmacytic lymphadenopathy; NOS, not otherwise specified.

We compared the CT findings obtained before and after treatment in the patients whose follow-up imaging was performed >6 months after their treatment initiation. At the timepoint of follow-up, all of the patients had achieved remission. The median interval between treatment initiation and follow-up imaging was 2.2 years (IQR 1.3–3.8 years). All six of the patients with TAFRO without iMCD/iMCD-TAFRO exhibited radiological improvement in anterior mediastinal abnormalities (**Figure 1B**), and a significant decrease in CT attenuation values was observed (p<0.05, **Figure 3A**). Conversely, only two of the four patients with iMCD-IPL/NOS showed radiological improvement (**Figure 1B**), and the changes in CT values were not significant (**Figure 3B**). A total of 21 patients also underwent CT-based evaluation of lymph nodes at intervals of more than 6 months, with improvement of lymphadenopathy observed in 15 patients (71.4%, **Supplementary Table S1**).

## Discussion

Our findings clarified that the CT imaging of anterior mediastinal lesions reveals distinct radiographic patterns that aid in differentiating TAFRO without iMCD/iMCD-TAFRO from iMCD-NOS and IgG4-RD. The non-mass-forming infiltrative pattern and the mass pattern were observed exclusively in TAFRO-related conditions, whereas the micro-nodular pattern was characteristic of iMCD-IPL/NOS and IgG4-RD. These anterior mediastinal abnormalities may serve as early imaging indicators of TAFRO syndrome. Our findings also highlight the diagnostic utility of CT attenuation values in distinguishing patients with TAFRO without iMCD/iMCD-TAFRO, those with iMCD-IPL/NOS, and those with IgG4-RD. The anterior mediastinal lesions in the present TAFRO patients demonstrated complete radiological resolution following treatment, whereas the lesions in patients with iMCD-IPL/NOS showed only partial improvement.

The key finding of this study is the significant difference in anterior mediastinal lesion patterns between patients with TAFRO-related conditions (iMCD-TAFRO and TAFRO without iMCD) and those with iMCD-IPL/NOS. Non-mass-forming infiltrative and mass-like lesions were almost exclusively observed in the TAFRO-related group, suggesting that these imaging patterns may be indicative of the hypervascular pathology characteristic of iMCD-TAFRO, as opposed to the lymphoproliferative features of iMCD-IPL/NOS (4). When comparing TAFRO-related disease with IgG4-RD, the distinction is even more evident. TAFRO syndrome is generally not characterized by marked elevations in serum IgG or IgG4 levels, and histologically, the lymph node architecture typically shows prominent vascular proliferation with relatively sparse plasmacytic infiltration (4). In contrast, IgG4-RD is a prototypical lymphoproliferative disorder characterized by dense lymphoplasmacytic infiltration, often accompanied by elevated serum IgG4 levels (18). Therefore, the marked differences in underlying disease biology likely account for the distinct imaging features, and few would dispute the radiographic dissimilarity between TAFRO-related conditions and IgG4-RD. In contrast, the comparison between iMCD-IPL/NOS and IgG4-RD revealed no statistically significant differences in CT attenuation values or morphological features. This result may reflect a shared pathophysiological basis, as both entities exhibit prominent lymphoplasmacytic infiltration and elevated immunoglobulin levels (4, 18). In this context, the inability of CT to distinguish between these two subtypes may be attributed to their similar cellular composition and tissue density. This observation emphasizes the need for integrating serological and histopathological data when differentiating between these conditions.

Other imaging studies revealed thoracic manifestations in TAFRO syndrome and iMCD, but few have focused specifically on anterior mediastinal lesions (12–14). A CT-based analysis of 13 cases of early-stage TAFRO syndrome highlighted various thoracoabdominal abnormalities, including a mild infiltration of anterior mediastinal fat; however, that study did not include a detailed classification or quantitative evaluation of these lesions (12). In an investigation of 11 patients with TAFRO syndrome, 64% of the patients showed an enlarged anterior mediastinum with a non-mass-forming infiltrative lesion (13).

A recent single-center study analyzing CT findings in 20 patients with iMCD reported that non-mass-forming infiltrative lesions in the anterior mediastinum were present in 67% (8 of 12) of patients with iMCD-TAFRO, whereas such findings were observed in only 13% (1 of 8) of patients with iMCD-IPL/NOS (14). This distribution is consistent with our present findings, which also demonstrated that non-mass-forming infiltrative lesions were predominant in the TAFRO without iMCD/iMCD-TAFRO group but rare in the iMCD-IPL/NOS group. However, unlike the previous study, which focused only on the presence or absence of anterior mediastinum lesions (14), our study evaluated CT attenuation values validated through ROC analyses, enabling accurate differentiations of TAFRO without iMCD/iMCD-TAFRO, iMCD-IPL/NOS, and IgG4-RD. Our study thus provides a more comprehensive diagnostic framework of differentiation through morphologic classification and quantitative imaging assessment. The diagnostic relevance of CT attenuation values suggests that incorporating anterior mediastinal lesion findings into current TAFRO or iMCD criteria may represent a potential strategy to improve diagnostic sensitivity and specificity.

The available case-based evidence highlights the variability of anterior mediastinal lesions in response to treatment, with indications that such lesions may evolve over the course of disease in TAFRO syndrome (7, 9, 12). In addition, although there is a case report describing a TAFRO patient who underwent debulking surgery for anterior mediastinal lesions (8), such a procedure was not observed in our patient population.

Our study has some limitations, including the restricted patient cohort. We also did not investigate the histological features of these lesions or the underlying mechanisms contributing to their development. Our study included IgG4-related disease as a control; however, it did not include comparisons with other diseases such as malignant lymphoma, which also can present with mediastinal lesions. Future research should focus on validating these findings in larger, diverse populations to enhance their generalizability and clinical applicability.

In conclusion, our findings highlight the significant diagnostic utility of CT attenuation values in differentiating TAFRO without iMCD/iMCD-TAFRO from iMCD-IPL/NOS and IgG4-RD. The findings advance our understanding of anterior mediastinal lesions as potential indicators for diagnosing TAFRO. This study demonstrates the impact of treatment on the progression of lesions in TAFRO patients, reinforcing the importance of the longitudinal assessment of imaging findings.

## Data Availability

The original contributions presented in the study are included in the article/**Supplementary Material**. Further inquiries can be directed to the corresponding author/s.
